# Working towards arabinogalactan proteins (AGPs) from fruit: carbohydrate composition and impact on fungal growth

**DOI:** 10.1186/s12870-022-04009-6

**Published:** 2022-12-20

**Authors:** Agata Leszczuk, Adrian Zając, Justyna Cybulska, Dawid Stefaniuk, Artur Zdunek

**Affiliations:** 1 Institute of Agrophysics, Polish Academy of Sciences, Doświadczalna 4, 20-290 Lublin, Poland; 2 Department of Functional Anatomy and Cytobiology, Institute of Biological Sciences, Maria Curie-Skłodowska University, Akademicka 19, 20-400 Lublin, Poland; 3 Department of Biochemistry and Biotechnology, Institute of Biological Sciences, Maria Curie-Skłodowska University, Akademicka 19, 20-400 Lublin, Poland

**Keywords:** Arabinogalactan proteins, Carbohydrate epitope, Cell wall, Fruit, Immunochemistry, *Penicillium notatum*, Proteoglycan

## Abstract

**Background:**

Arabinogalactan proteins (AGPs) are extracellular matrix constituents involved in plant response to fungal infection. The aim of the current study was to investigate the antifungal effect of AGPs ex situ and to determine the structural features of AGPs that may have an influence on this activity. The features of AGPs isolated from fruit were investigated with molecular tools based on specific monoclonal antibodies recognizing carbohydrate AGP epitopes. The Antifungal (well-diffusion) Susceptibility Test and the Agar Invasion Test were used to assess the impact of AGPs on *Penicillium notatum* culture.

**Results:**

The results definitely ruled out the influence of AGPs on fungal growth. The immunochemical analyses revealed that AGPs consist mainly of carbohydrate chains composed of β-linked glucuronosyl residues recognized by LM2 and GlcA-β(1 → 3)-GalA-α(1 → 2) Rha recognized by JIM13, which do not have the same functional properties outside the plant cell in in vitro experimental conditions.

**Conclusions:**

The action of a single cell wall component does not elicit any influence ex situ*.* The extensive accumulation of glycan chains of AGPs in infected tissue as a result of a complex mechanism occurring in the cell wall emphasizes the importance of dependencies between particular components of the extracellular matrix in response to fungal attack.

**Supplementary Information:**

The online version contains supplementary material available at 10.1186/s12870-022-04009-6.

## Introduction

Arabinogalactan proteins (AGPs) are plant extracellular matrix components [1]. According to current knowledge, AGPs are classified as cell wall proteins (CWPs) involved in signaling [2]. Due to their structure, these highly glycosylated AGPs represent the superfamily of hydroxyproline (Hyp)-rich O-glycoproteins HRGPs [3]. AGP sugar chains account for over 90% of the total AGP molecular mass. The polysaccharide units vary in length (30–150 sugar residues) but retain the structure of arabinogalactan type II (AG II). The side chains can be further modified with arabinose and other sugars, including L-rhamnose, L-fucose, D-glucosamine, D-mannose, D-xylose, D-glucose, D-glucuronic acid, and D-galacturonic acid [3]. Extensive glycosylation exerts direct effects on AGP functions [4, 5]. One of the basic roles of AGP is the crosslinking between other cell wall polysaccharides like rhamnogalacturonan I and hemicellulosic arabinoxylan in a structure named the APAP1 complex [6, 7]. Moreover, the spatial and temporal distribution of AGPs is correlated with their molecular features. AGPs are attached to the plasma membrane by a glycosylphosphatidylinositol (GPI anchor). In turn, their carbohydrate chains are mainly located in the cell wall compartment [8]. Nowadays, it is assumed that, in the presence of phospholipase C (PLC) and phospholipase D (PLD), AGPs can be cleaved and released from the plasma membrane into the cell wall and the extracellular matrix [1]. The interactions between the components and the assembly at the cell wall-plasma membrane border facilitate continuous flux throughout the entire extracellular matrix during various processes.

In the first report on the role of AGPs during the ripening process, Fragkostefanakis and coworkers [9] described the changeable expression of AGPs, which indicates its possible regulation by the developmental program of ripening. Also, the authors noted similar patterns of *SlAGP4* and *LeAGP1* gene expression in fruit ripening and mechanical wounding, indicating involvement in abiotic stresses. The promoter analysis provided information on the transcriptional regulation of both genes, which suggested AGP function in response to mechanical wounding [9]. Our previous studies on AGPs in fruit conducted using immunocytochemical techniques and imaging at the cellular level (CLSM) and the subcellular level (TEM) revealed tissue specificity [10] and the presence of various carbohydrate epitopes of AGPs in the cell wall-plasma membrane continuum according to a strict pattern [11, 12]. AGPs extracted from fruit at different stages of the growth and ripening process are characterized by variable monosaccharide content and molecular mass [13]. Also, microscopic imaging with SEM and AFM approaches showed the ability of AGPs to form numerous aggregates composed of many significantly smaller individual molecules [14]. All these features are correlated with particular phases of the developmental program in fruit, which are indisputably related to the degradation of sugar chains in the AGP molecule.

It is known that fungal disease is closely associated with intercellular communication between the plant cell and the microbe, with a strong emphasis on the role of the cell wall in mechanical support [15]. However, the cell wall is not only a passive barrier against pathogens. The defense role of the cell wall consists in triggering molecular mechanisms of sensing stress conditions [16]. Activation of enzymes necessary for the hydrolysis of carbohydrates, e.g. pectinases inducing pectin depolymerization, is one of the causes of morphological changes in the fruit structure during plant-microbe interactions [17]. Destruction of cross-links between cell wall polysaccharides and pectins through degradation of arabinogalactans (AGs) by endo-β-(1,3), β-(1,4), and β-(1,6)-galactanases, α-L-arabinofuranosidases, β-L-arabinopyranosidases, and β-D-glucuronidases induces disruption of cell wall integrity [18]. Interestingly, in situ investigations on the distribution of AGPs in fruit tissue during fungal disease caused by *Penicillium spinulosum* invasion allow correlating the extensive accumulation of AGPs in randomly degraded surfaces filled by fungal spores. Moreover, with the progress of fungal infection in damaged tissue, AGP epitopes were noted also in cytoplasm compartments, indicating that the cellular arrangement of AGP epitopes is spatially regulated within the extracellular matrix during the fruit disease caused by *Penicillium spinulosum* [19]. Together, these results show that AGPs with other cell wall components may create a barrier impermeable to fungal spores to stop any progressive infection in fruit. The abundant localization of AGP epitopes in disorganized tissue underlined the potential involvement of AGPs in the cellular response to stress conditions [19].

Taking into account our previous investigations on AGPs in fruit [19] and the well-known function of arabinogalactan type II degradation in plant-microbe interactions [18], in this paper, we described the effect of AGPs on *Penicillium* species. In this study, we used cultures of *Penicillium notatum*, which infect fruit causing damage both during cultivation and after harvest. The general aim of the current work was to determine the potential effect of AGPs on fungal activity ex situ. To determine the impact of AGPs on fungal growth, two microbiological assays were performed. Next, to elucidate the observed results, immunochemical analyses of the AGP glycan structure were performed using molecular approaches with well-defined antibodies against AGPs.

Moreover, based on the knowledge about the variability of the AGP structure during the ripening process, mainly the carbohydrate chains, an attempt was made to examine whether changes in the glycan content affect the growth of the *Penicillium.* Both, our previous observations of the cellular distribution of AGPs in fruit undergoing fungal infection and the current study allow concluding that the interactions between all cell wall constituents are responsible for crucial changes in the cell wall assembly during a pathogen attack.

## Material and methods

### Plant material

Apple fruit cv. ‘Jonaprince’ were provided by a local producer (Lublin, Poland). The apple fruit were collected before the ripening process (Immature Green) at the optimum harvest date for this cultivar (Mature Red) and after 1 month of storage (Stored). Then, fruit (10) of each stage with similar features and without visible symptoms of the disease were chosen for the experiments. The stages of ripening were selected and characterized in our previous paper [14].

All methods were performed in accordance with the relevant guidelines and regulations. The datasets used and analyzed during the current study are available from the corresponding author upon reasonable request.

### Isolation of AGPs using Yariv reagent

The isolation of AGPs was carried out according to Lamport’s procedure [20] with small modifications described in our previous papers [13, 14]. Briefly, frozen homogenized apple parenchymal tissue was mixed in 2% CaCl_2_ for 3 h at room temperature (RT). Next, the sample was stirred for 30 min (centrifugation at 10000 *x g*) and left for precipitation with Yariv reagent (β-GlcY; commercially available from Biosupplies, Australia) overnight at RT. Yariv reagent [1,3,5-tri(p-glycosyloxyphenylazo)-2,4,6-trihydroxybenzene] is a useful tool for staining and detection of AGPs by binding to the β-(1–3)-linked D-Galp backbone of AGPs. The specific interaction of AGPs with Yariv reagent forming a brown-red precipitate has been defined as a significant criterion in the characterization of AGPs [21, 22]. Subsequently, the precipitate was collected by centrifugation for 10 min (2000 *x g*) and resuspended in distilled water. For reduction of the diazo linkage, sodium metabisulfite (Thermo Fisher, Germany) was added gradually and the sample was heated until the solution became clear yellow. The supernatant was transferred to a dialysis bag with a 12-kDa MW cutoff (32 mm flat width, Sigma, USA) and dialyzed for 24 h with three changes of water. Finally, the dialysate was freeze-dried.

### Antifungal (well-diffusion) susceptibility test (AFST)

The isolates of AGPs were tested for potential in vitro antifungal susceptibility using the AFST and AIT assays with the method proposed by Pinto [23] with modifications. Wells with a diameter of 6.9 mm were made in Petri dishes with Mueller-Hinton agar medium. The plates were inoculated with a spore suspension with a final CFU of 2 × 10^^4^. The inoculum was prepared from a 7-day-old sporulating culture of *Penicillium notatum* grown on GPY media by scraping the spores into 1 mL of sterile water and making a suspension with an optical density corresponding to 1 McFarland standard unit. The exact CFU was determined by dilution and colony counting. 100 μL of the solutions with the final 25–500 μg content of the tested substance were added to each well, and a Gum Arabic (Biosupplies, Australia) solution was used as a control. It is well known that AG chains comprise as much as 90–98% of Gum Arabic, and the second major fraction of Gum Arabic has been identified as AGPs [24, 25]. The tests were performed in triplicates. The results were read after 48 hours of growth at 26 °C.

### Agar invasion test (AIT)

Both the inoculum and the medium were prepared as in the AFST assay. A solution of the tested substance in sterile water with a final concentration of 1000 μg/mL mixed 1:1 with Mueller-Hinton Agar (kept at 55 °C in a water bath) was added to 20 mm diameter wells. Afterward, the process of mycelium invasion from the outer perimeter of the well to its interior was observed for 5 days at 26 °C. Gum Arabic (Biosupplies, Australia) with the same concentration as the tested samples was used as a positive control. The results are presented as the percent difference between the diameter of the mycelial invasion in the test sample and the control.

### Purification - separation of AGP fractions

Freeze-dried AGPs were dissolved in 0.1% trifluoroacetic acid (TFA) and centrifuged at 9000 *x g*. The supernatant was analyzed using HPLC as previously described by Gao [26]. RP-HPLC was carried out with a Hamilton PRP-1 column (250 mm, 4.6 mm internal diameter) using a system consisting of an 1130 HPLC quaternary pump, an S 5300 sample injector, an S 4120 column oven, and an S 3350 PDA Detector (Sykam GmbH, Gewerbering, Germany). The sample was eluted and collected with gradient solvents: A (0.1% TFA) and B (0.1% TFA in 80% acetonitrile). Following the method proposed by Gao [26], the gradient elution included 100% A for 1 min, then up to 40% B for 100 min, followed by a return to 100% A for 110 min. The flow rate was 0.5 mL/min, the temperature of the separation was 25 °C, and the detection wavelength was 220 nm. According to molecular weight, three major fractions (1–3) were collected and freeze-dried.

### Yariv reagent reactivity - radial gel diffusion assay

For rapid detection and semi-quantification of AGPs, the radial gel diffusion assay using Yariv specific reactivity was carried out as in Holst and Clarke [27] and Castilleux [28]. The samples, i.e. AGP fractions (1–3), were applied to a gel plate containing 0.002% (w/v) Yariv reagent (β-GlcY, Biosupplies, Australia), 0.15 M NaCl, 0.02% (w/v) sodium azide, and 1% (w/v) agar. The samples (40 μL per well) were loaded as water solutions (2 mg/mL) and incubated for 48 h at RT. The relative reactivity of β-GlcY was quantified using Gum Arabic (Biosupplies, Australia) as a standard (positive control). Distilled water was used as a negative control. For quantitative analysis of the reactivity of the β-GlcY/AGP concentration, the halo areas around the wells were manually identified and counted with ImageJ 1.51 software (https://imagej.nih.gov/). The assay was performed in triplicate.

### Validation of AGPs presence in fractions - immuno-dot blot assay

Three selected fractions of AGPs were validated by the dot blot assay [6]. The fractions were diluted in SDS buffer: 50 mM Tris-HCl, pH 6.8, 2% SDS, 10% glycerol, 5% β-mercaptoethanol (710 mM), and 12.5 mM EDTA. Each sample (80 μg/mL) was dotted onto an Immmobilon P-PVD transfer membrane with a 0.45 μm pore size (Millipore IPVH00010). The samples were air dried, then membranes were incubated with methanol within 5 min and the dots were subsequently blocked with a 5% BSA blocking solution (Sigma, USA) in PBS, pH 7.4 (Sigma, USA) for 1 h at RT. PBS was prepared in accordance with the manufacturer’s protocol. One tablet dissolved in 200 mL of deionized water yielded 0.01 M phosphate buffer (pH 7.4), 0.0027 M potassium chloride, and 0.137 M sodium chloride at RT. After washing with PBS, the samples were incubated with primary antibodies diluted 1:100 in 1% BSA in PBS for 2 h at RT. For the detection of AGPs, monoclonal antibodies recognizing specific epitopes of the AGP glycan moiety were used (Table 1). The membrane was washed in PBS, and the samples were incubated with secondary anti-rat antibodies conjugated with alkaline phosphatase (anti-rat-IgG, Sigma, USA) diluted 1:1000 in 1% BSA in PBS for 2 h at RT. After immunochemical reactions, the membrane was washed in PBS, and AGPs were detected with AP substrates: 5-bromo-4-chloro-3-indolylphosphate (BCiP, Sigma, Switzerland) and Nitrotetrazolium (NBT, Sigma, USA) in N, N-dimethylformamide (DMF, Thermo Fisher, Germany). All the analyses of the samples were performed in triplicate.

**Table 1:** Monoclonal antibodies against AGPs with their specificity

Antibody name	Specificity of AGPs epitopes	Reference
JIM13	GlcA-β(1 → 3)-GalA-α(1 → 2)Rha	[29]
JIM15	GlcA-β(1-*O*-Me)	[29]
LM2	β-D-GlcA	[30]
LM14	AG type II	[31]

### Molecular characterization of AGPs - SDS-PAGE gel electrophoresis and Western blot detection

For detailed identification of the molecular mass of each AGP fraction, a Western blot with prior electrophoresis was carried out according to our previous studies [14] with slight modifications. Particular fractions of AGPs were dissolved in SDS buffer, boiled at 99 °C for 5 min, and centrifuged for 10 min. The samples (25 μL per lane) were separated by 8% SDS-PAGE. Upon completion of electrophoresis, gels with proteins were transferred onto an Immmobilon P membrane with 0.45 μm pore size (Millipore IPVH00010). First, the membranes were blocked with UltraCruz® Blocking Reagent (SantaCruz Biotechnology) for 1 h at RT, and then incubation with appropriate primary antibodies at a concentration of 1:100 was carried out overnight at 4 °C. After the incubation, the membranes were washed with PBS buffer enriched with 0.05% Triton X-100 (Sigma, USA). Next, the membranes were incubated with secondary anti-rat antibodies conjugated with alkaline phosphatase (Sigma, USA) for 2 h at RT. Finally, immunoreactive bands were visualized with AP substrates (BCiP and NBT). Gum Arabic (Biosupplies Australia, concentration 1:1000) was a positive control. The results of the dot-blot assay, as well as Western blot, were imaged using the GelDoc Go Imaging System (Bio-Rad, USA). The quantitative analysis of protein bands was performed using Image Lab Software, version 6.1 (Bio-Rad, USA).

### Molecular characterization of AGPs – enzyme-linked immunosorbent assay (indirect ELISA test)

The estimation of the binding of four different monoclonal anti-AGP antibodies JIM13, JIM15, LM2, and LM14 (Kerafast, USA) was evaluated in the control (Gum Arabic) and tested samples as proposed by Pfeifer [32]. First, 96-well plates (Nunc MaxiSorp™ flat-bottom, Thermo Fisher, Denmark) were preincubated and coated with different concentrations of AGPs, Gum Arabic, and three fractions (0, 25, 50, 100 μg mL − 1 in distilled water, 100 μL per well) at 37.5 °C with open cover with constant shaking for 72 hours. Then, the plates were washed three times with PBS buffer (pH 7.4 with 0.05% Tween 20) and blocked with 0.1% BSA in PBS (pH 7.4, 100 μL per well, 1 h at 37.5 °C). The blocked plates were washed again with the same washing buffer as described above. Next, 100 μL of primary antibodies diluted at 1:20 was added to each well and the plates were incubated for 1 h at 37.5 °C followed by a three-fold washing step. The secondary antibody anti-rat-IgG conjugated with alkaline phosphatase (Sigma, USA) was pipetted in a dilution of 1:500 in PBS. After the incubating and washing steps, color was developed through the addition of 1-Step™ PNPP p-nitrophenyl phosphate disodium salt (100 μL per well, Thermo Fisher, USA). According to the Thermo Fisher instructions, the water-soluble yellow reaction product absorbs light at 405 nm. After incubation for 20 minutes at RT, the reaction was stopped with 2 N NaOH (50 μL well− 1). The absorbance of each well was measured in an ELISA reader (MPP-96 Photometer, Biosan) and analyzed with Quant Assay Software. The samples were tested in triplicate. The data were statistically analyzed using Statistica v.13 (TIBCO *Software* Inc. USA), an analysis of variance (one-way ANOVA) followed by post hoc Tukey’s honestly significant difference (HSD) test was used, with the significance level estimated at *p* < 0.05.

## Results

The extraction with Yariv reagent yields AGPs, which are characterized by a unique composition. In the current study, AGPs isolated from apple fruit at three stages of ripening were used to investigate their hypothetical effect on *P. notatum* growth. Next, molecular approaches were used to elucidate the results.

### Effect of AGPs on *P. notatum* growth

**Fig. 1:**
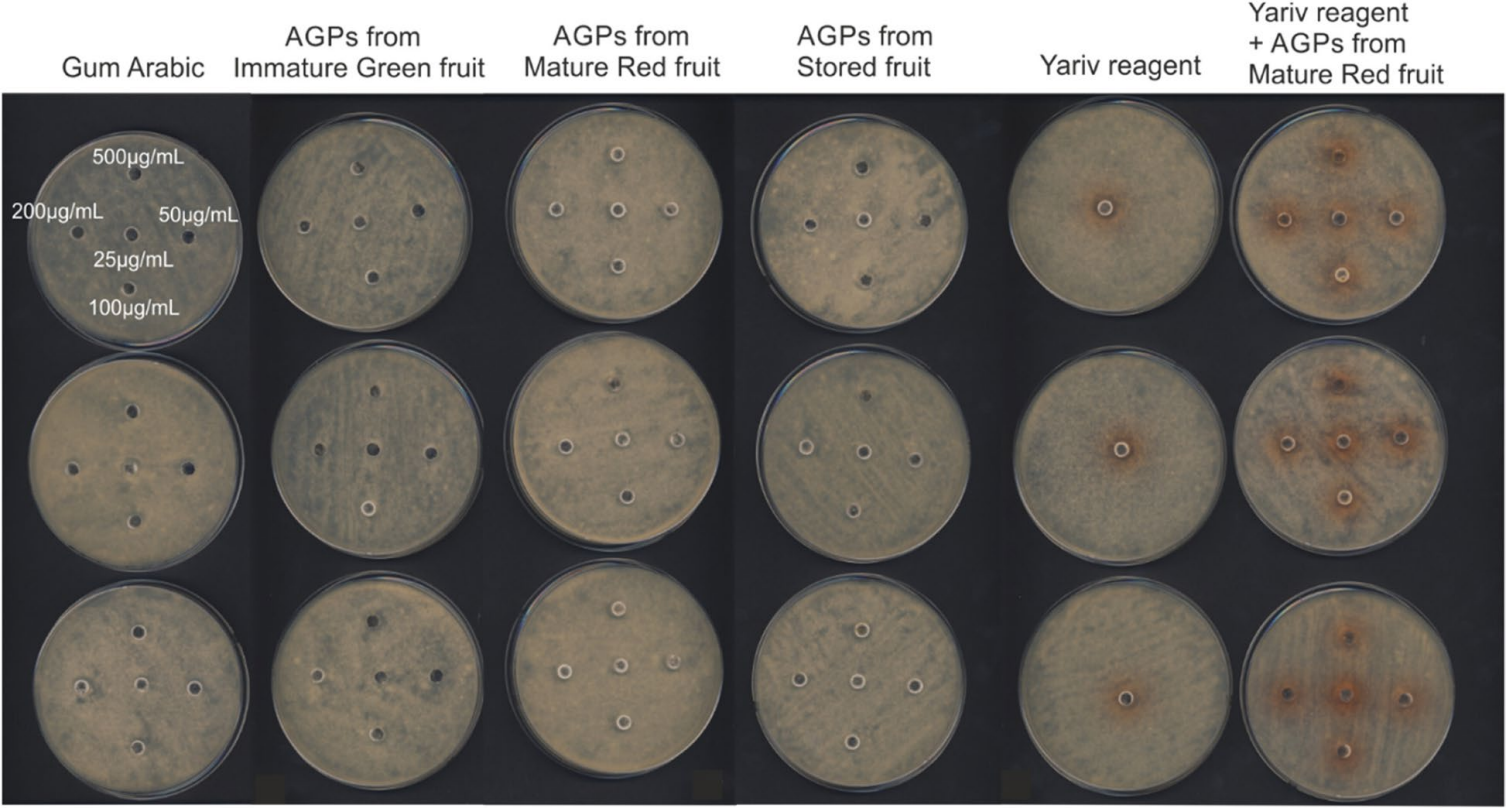
Antifungal (well-diffusion) Susceptibility Test (AFST). *P. notatum* culture with the addition of AGPs from Immature Green fruit, Mature Red fruit, and Stored fruit as well as Yariv reagent and mix of Yariv reagent and AGPs from Mature Red fruit. The test was performed for the final substance concentration of 500 μg (upper well), 200 μg (left well), 100 μg (lower well), 50 μg (right well), and 25 μg (central well) of the tested substance and Gum Arabic as a control (first column). The experiment showed no changes in *P. notatum* growth in the three repetitions (rows)

In order to determine the effect of AGPs obtained from apple fruit, Yariv reagent, a mixture of Yariv and AGPs from mature red apple, and Gum Arabic (as control) on germination of *P. notatum*, a well-diffusion test was performed with the final substance’s concentration of 500 μg (upper well), 200 μg (left well), 100 μg (lower well), 50 μg (right well), and 25 μg (central well) (Fig. 1). There was no visible inhibition of microbial growth by any tested substance, regardless of the concentration of the substance and the maturity of the apple used for AGPs extraction. Also, the Yariv reagent and the mix of AGPs with the Yariv reagent did not exert an effect on the growth of *P. notatum.* Furthermore, the experiment with Gum Arabic as a positive control yielded the same result. This may have been related to the poor diffusion of the glycoprotein into the agar, which is why the AIT test was performed. Minor differences in the inside-well growth of fungi were caused by physical limitations of the spores distribution on plates.

**Fig. 2:**
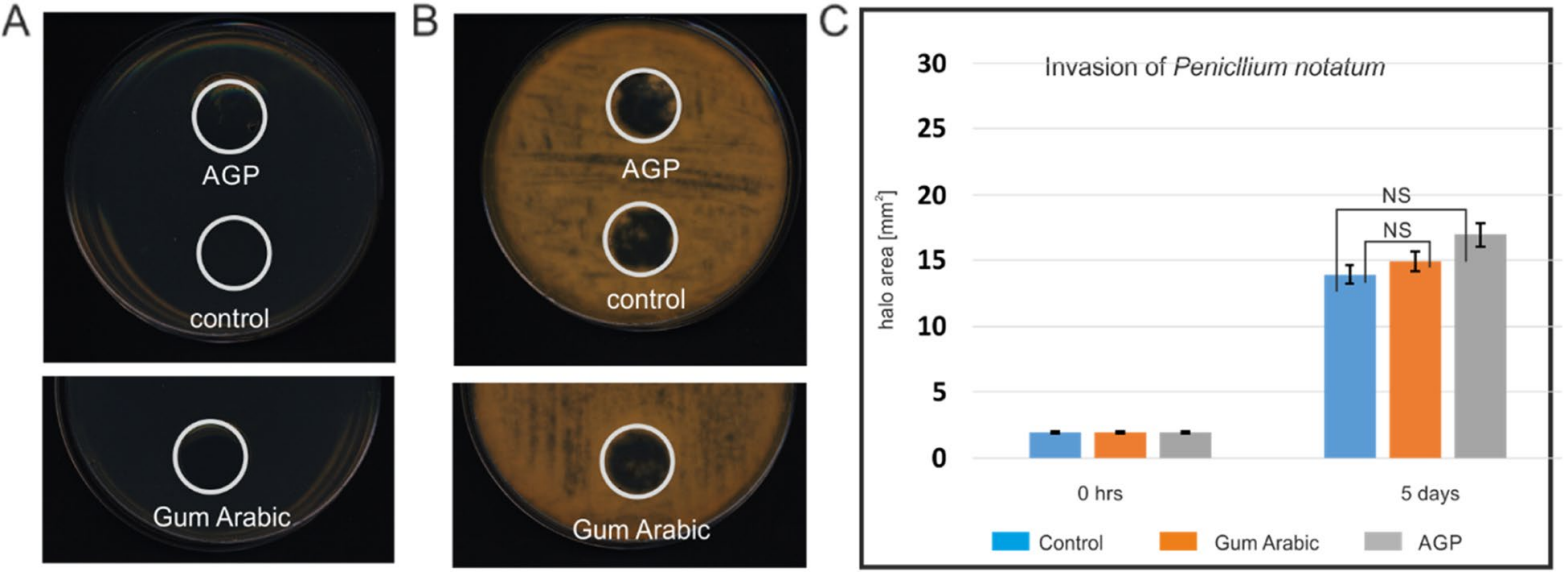
Agar Invasion Test (AIT). Test with AGP, control, and Gum Arabic at the start of the assay at 0 hrs a and after 5 days of growth b. The quantitative analysis of the results confirmed the absence of an effect on *P. notatum* growth. No statistically significant results were obtained c. The experiment was carried out in three replications. Abbreviation: NS – Not Statistically Significant

To verify this hypothesis, an agar invasion test (AIT) was performed, in which the same substance was dissolved in the agar medium, and the potential growth of fungi in the tested area was observed (Fig. 2). AIT has confirmed no effect of the studied substances on fungal growth. Also, no statistically significant differences between AGP and the control (pure medium) and Gum Arabic were observed (Fig. 2c).

### Identification of AGPs extracted from fruit

**Fig. 3:**
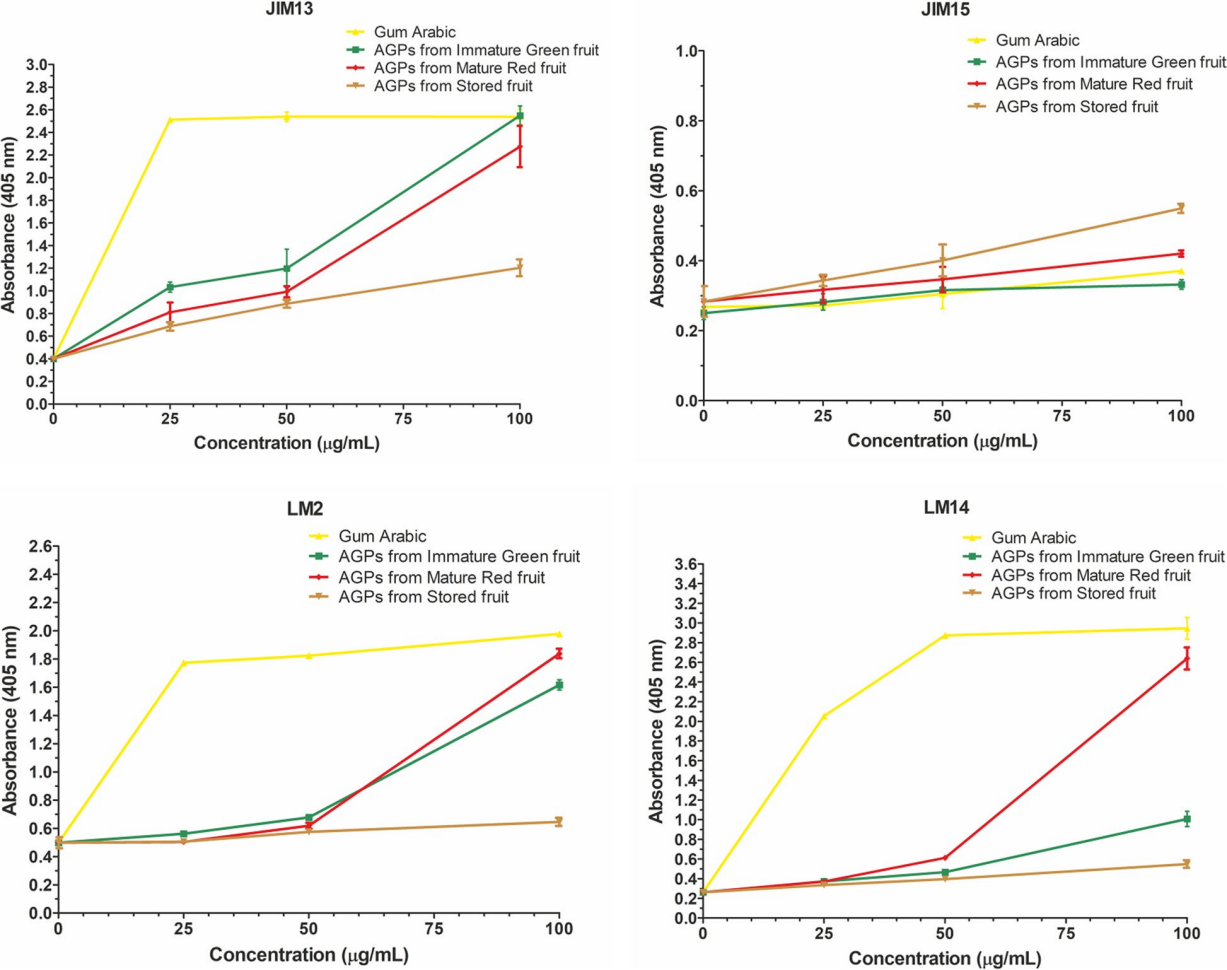
ELISA test with JIM13, JIM15, LM2, and LM14. AGPs from Immature Green fruit, Mature Red fruit, and Stored fruit. Gum Arabic as a positive control. The ELISA test was performed in triplicate

For the identification and characterization of AGPs isolated from the apple fruit at different stages of ripening, the ELISA test with antibodies against AGPs was performed (Fig. 3). The most often applied commercial antibodies JIM13, JIM15, LM2, and LM14 were chosen. Our previous studies conducted with the use of the same types of antibodies have shown the localization of these epitopes in fruit in situ [10–12, 19]. Qualitative data of ELISA tests either confirm and/or denies whether the presence of particular epitopes in samples of extracted AGPs from fruit at different stages of ripening. In the present experiment, the ELISA test revealed the presence of JIM13, LM2, and LM14 epitopes ex situ*.* None of the analyzed samples showed reactivity with JIM15. Thus, the presence of JIM15 epitopes was excluded. In the AGPs isolated from the Immature Green fruit, the highest level of the GlcA-β(1 → 3)-GalA-α(1 → 2) Rha epitope recognized by JIM13 was noted. In turn, in the AGPs isolated from the Mature Red fruit, the AG type II epitope recognized by LM14 noticeably predominated. Quantitative analysis of the absorbance allows underlining that samples of AGPs from all examined stages of ripening do differ based on the concentration of LM2 and LM14 epitopes. Also, in the case of LM2 and LM14, the results mark significant statistical differences in tested samples from mentioned stages of the ripening process (Fig. 3 and Supplementary Material).

### Purification and detailed characterization of AGPs fractions

**Fig. 4:**
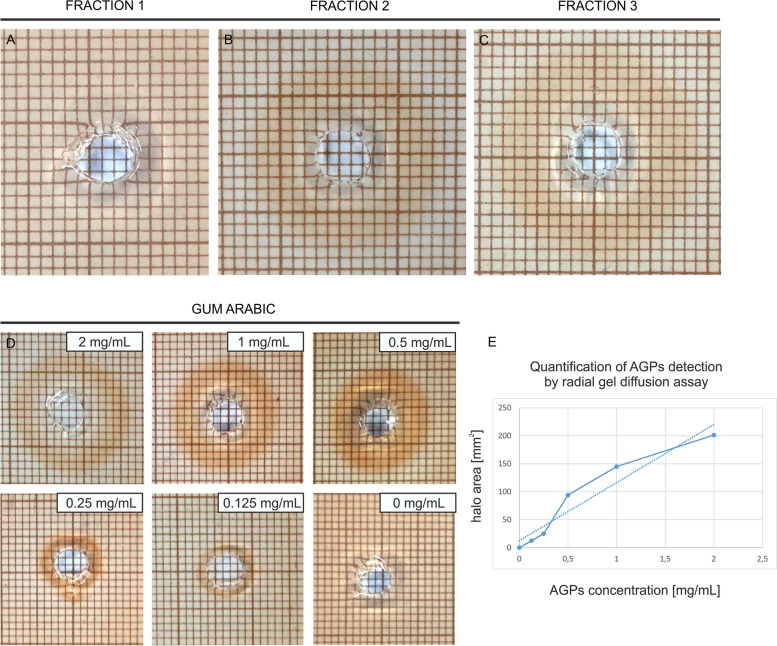
Detection of AGPs by the radial gel diffusion assay in fraction 1 a, fraction 2 b, and fraction 3 c. Serial dilutions of the standard Gum Arabic solution: 2 mg/mL, 1 mg/mL, 0.5 mg/mL, 0.25 mg/mL, 0.125 mg/mL, 0 mg/mL d, and quantification of AGP detection e. The analyses were performed in triplicate

**Fig. 5:**
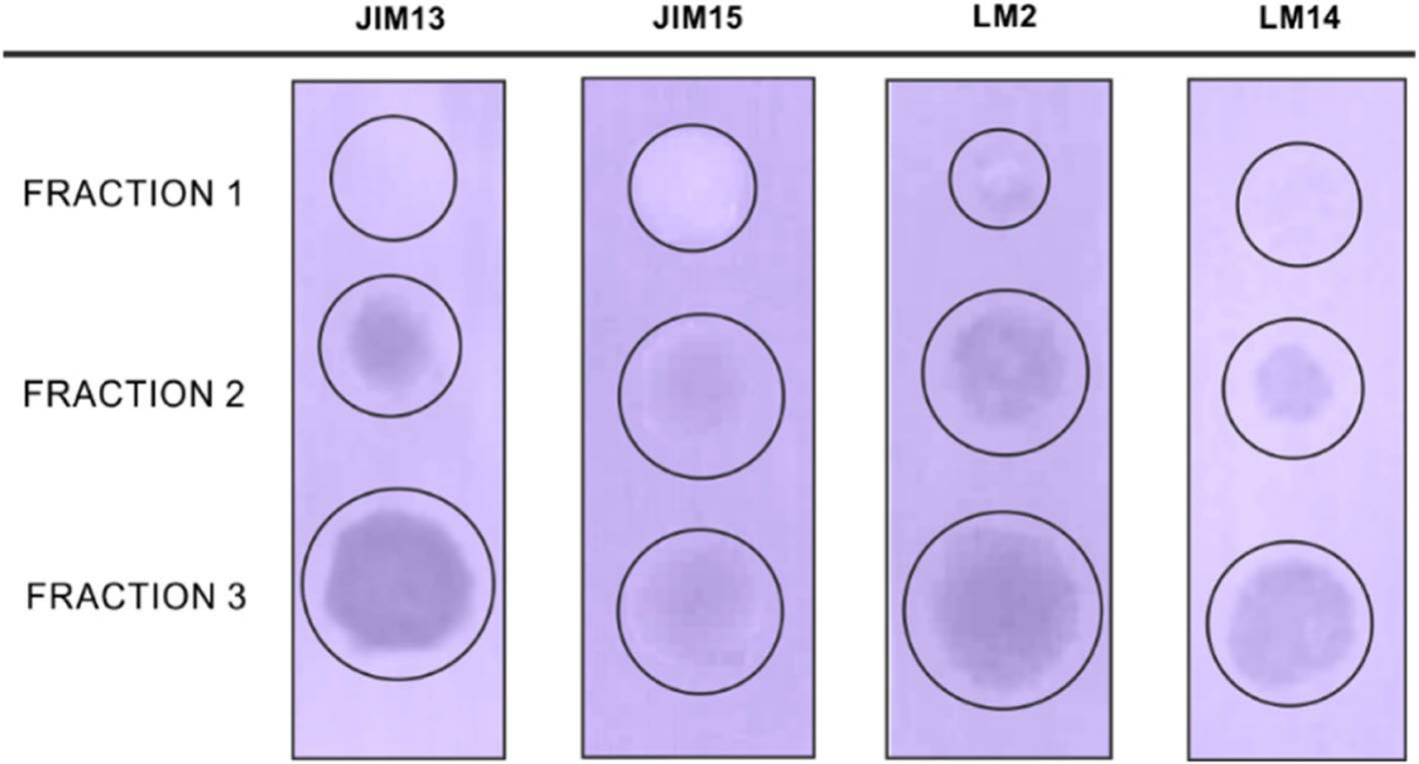
Immuno-dot blot analysis of AGP fractions 1, 2, and 3 using JIM13, JIM15, LM2, and LM14 antibodies. The visible colorful dots confirm the presence of AGP-specific epitopes in the analyzed fractions. The analyses were performed in triplicate

AGPs extracted from the fruit and lyophilized were separated into fractions using HPLC. The separation allowed the selection of three major fractions, referred to as fraction 1, fraction 2, and fraction 3 in this experiment. To confirm the presence of AGPs in the three fractions, two methods based on the use of distinctive markers against AGPs were employed: radial gel diffusion using Yariv reagent specific reactivity (Fig. 4) and immno-dot-blotting with specific monoclonal antibodies recognizing AGP epitopes (Fig. 5).

The qualitative and quantitative results of the radial gel diffusion assay are shown in Fig. 4. A positive reaction indicating the presence of AGPs is seen as a ring diffusing from the well, and the lack of the visible halo indicated the absence of specific reactivity. The radial gel diffusion assay confirmed the presence of AGPs in two fractions: fraction 2 (Fig. 4B) and fraction 3 (Fig. 4C) and proved the absence of AGPs in fraction 1 (Fig. 4A). According to the relative reactivity of Yariv reagent (β-GlcY) and using Gum Arabic as a standard (Fig. 4D and E), the concentration of AGPs was quantified in fraction 1 (0 mg/mL), fraction 2 (0.98 ± 0.23 mg/mL), and fraction 3 (1.55 ± 0.12 mg/mL). Based on the results obtained, the AGP concentration in fraction 1 may be much lower, but it is not possible to calculate it using the gel diffusion assay.

Also, the three fractions of AGPs were characterized in terms of the binding capacity of several specific monoclonal antibodies. Antibody binding was assessed with the dot-blot method (Fig. 5). The same antibodies as in the ELISA test were used: JIM13, JIM15, LM2, and LM14. The colored dots on the membrane indicated samples that were positive for the target AGP proteins. Also, the first evident observation is the most intensive reaction of fraction 3 with all the antibodies used. Only JIM15 gave a significantly less intensive reaction, which may suggest that only a trace amount of the epitope detected by that antibody was found in tested samples with the smallest detection in fraction 1. The stronger dot intensity and the higher dot size revealed differences in the AGP content in the analyzed fractions. The largest halo area and the most visible color reaction of fraction 3 correspond to the results obtained in the radial gel diffusion. This allows us to conclude that fraction 3 is the most abundant in AGP epitopes. The effect of the reaction with fraction 2 is similar; however, the dots are less visible than in the case of all the antibodies used. This also proves the lower content of epitopes recognized by the antibodies than in fraction 3.

**Fig. 6:**
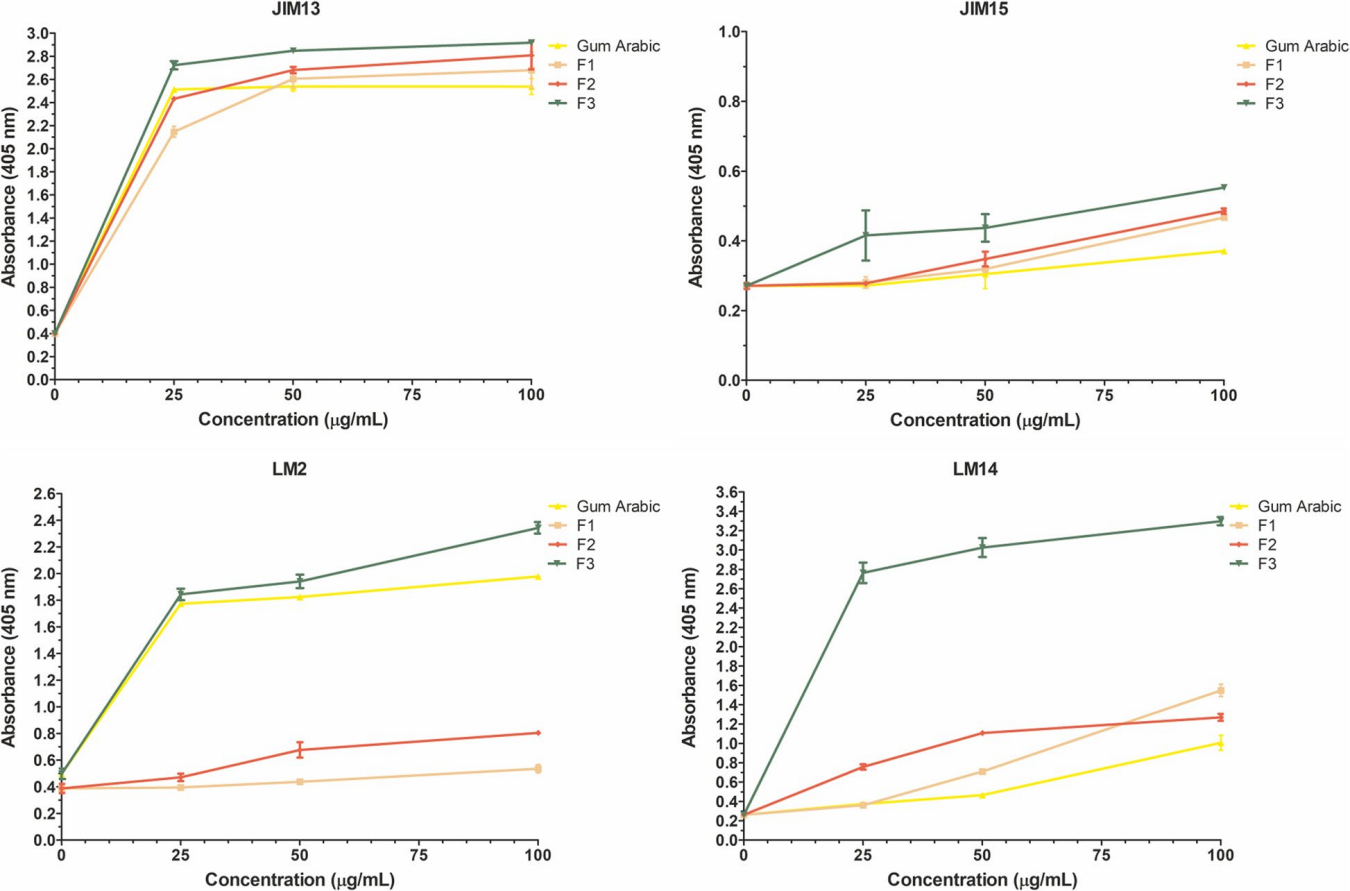
ELISA test of fraction 1 (F1), fraction 2 (F2), and fraction 3 (F3). AGPs were detected by JIM13, JIM15, LM2, and LM14 antibodies. Gum Arabic was used as a positive control. The ELISA test was performed in triplicate

The ELISA method was used to test the reactivity of the antibodies against the AGP fractions (Fig. 6). All the fractions reacted with JIM13 with visible differences at a concentration of 25 μg/mL. A similar result was obtained with JIM15, but the absorbance was very low, indicating the absence of JIM15 epitopes in all the fractions which correspond with the results obtained by dot-blot assay. Interestingly, the interactions with LM2 and LM14 underline the strong binding to fraction 3 (significantly higher compared to control and other fractions). Also, the significantly high level of absorbance with LM14 indicates that fraction 3 is composed mainly of AG type II. Moreover, statistical analyses allow finding differences between all examined fractions, also in comparison to Gum Arabic. Among all fractions, fraction 3 was distinguished by the highest content of epitopes recognized by the used four antibodies (Fig. 6 and Supplementary Material).

**Fig. 7:**
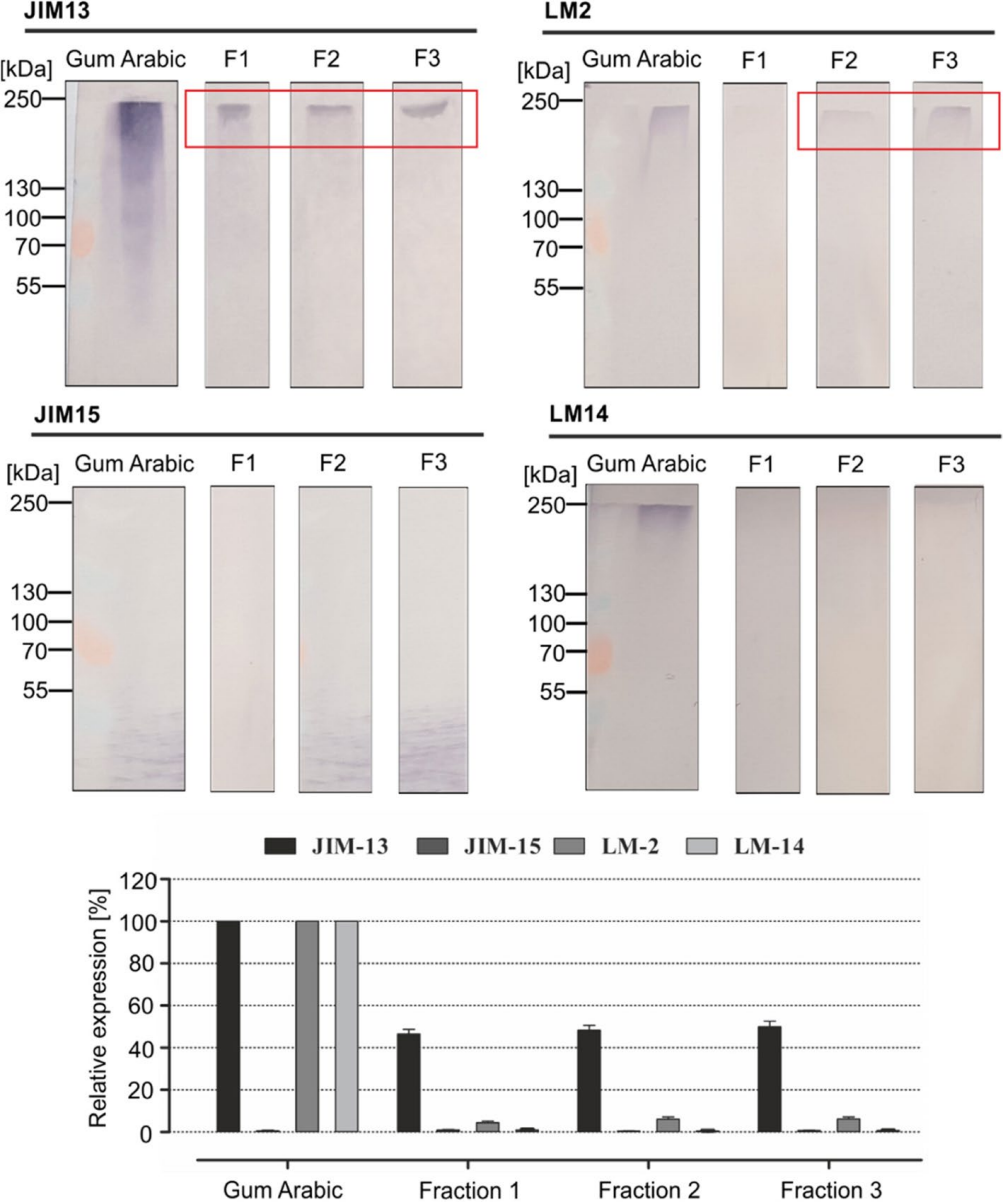
SDS-PAGE and Western blot analysis of fraction 1 (F1), fraction 2 (F2), and fraction 3 (F3). AGPs were detected by JIM13, JIM15, LM2, and LM14 antibodies. Both, the electrophoresis and Western blot were performed in triplicate

The bands on the membranes after the electrophoresis and Western blot (Fig. 7) partly correspond to the dots visible in the dot blot analyses (Fig. 5). The results of the molecular studies, including SDS-PAGE and immunoblotting techniques, also confirm the presence of specific epitopes recognized by JIM13 and LM2 in the AGP fractions. However, after electrophoretic separation, AGPs were not detected by JIM15 and LM14 antibodies in any of the fractions. Also, the bands visible after detection using JIM13 were characterized by a high molecular mass of around 250 kDa for molecules in all the fractions. Western blotting with LM2 antibody revealed the presence of AGPs in fraction 2 and fraction 3, with similar molecular mass as in the analyses with JIM13.

## Discussion

Studies on fruit tissue have demonstrated the correlation between AGPs distribution, molecular features, and role in the development and ripening process [33]. The results of our previous research shown also an extensive accumulation of AGPs in fruit tissue as a response to fungal infection [19]. Those experiments prompted subsequent work to test the AGP effect on *Penicillium* growth ex situ presented in this experiment composed of three parts: (1) microbiological assays, (2) analyses confirming the presence of AGPs in the fractions of AGPs-rich isolates, separated according to their molecular weight, and (3) description of their molecular properties and glycome profiling, which may be an explanation of the lack of interaction between AGPs and pathogens.

Early, studies performed by Sun and coworkers [34] on the characterization of AGPs from tomato revealed the occurrence of two major peak fractions and three minor peak fractions. Subsequent analyses showed that only one fraction contained AGPs with a molecular weight of about 48 kDa [34]. Electrophoretic profiling and immunocytochemical detection of AGPs in olive during the embryological process carried out by Castro and coworkers [35] demonstrated the presence of AGPs with molecular weights ranging from 90 to 120 kDa and a ca. 318 kDa band. In the present work, carbohydrate epitopes were detected in all the Yariv reagent precipitates irrespective of the stage at which the fruit AGPs were extracted. Thus, the fractionation of the Yariv reagent precipitate by HPLC allowed clear separation and purification of AGPs. Then, the dot blot assays, and immunoprinting on nitrocellulose membrane revealed the presence or absence of AGPs in the fractions obtained. The content of AGPs was quantified by radial gel diffusion and, based on the use of Gum Arabic as a standard, it was revealed that fraction 2 contained 0.98 ± 0.23 mg/mL of AGPs, and fraction 3 was characterized by 1.55 ± 0.12 mg/mL of AGPs. After the purification and separation of AGPs, the ELISA test detected β-linked glucuronosyl residues of AGPs recognized by LM2 and a typical GlcA-β(1 → 3)-GalA-α(1 → 2) Rha trisaccharide recognized by JIM13 in the two main fractions. Additionally, to provide more information, the AGP fractions were separated by SDS-PAGE electrophoresis, blotted, and probed with antibodies. The JIM13 antibody reacted very strongly with a single band, which indicated the molecular weight of 250 kDa of AGPs in all the fractions. These results are untypical because the Western blot of Yariv reagent precipitates usually shows smeared material [9]. This was confirmed by the detection of a single sharp band at about 250 kDa with the use of the LM2 antibody. Thus, the presence of various glycan epitopes recognized by monoclonal antibodies and the different Yariv reagent reactivity allows for identifying the heterogeneous occurrence of AGPs in the fractions. The data presented in the current study confirm the significant potential of glycome profiling and immunochemical assays in the detection and characterization of AGPs, with an emphasis on the determination of structural changes in the fruit ripening program. The detected binding affinities are similar to those reported in previous studies on the degradation of AGP sugar chains during the ripening process [13, 14]. Also, these results correspond to the basic information about the binding capacity of the Yariv reagent. It is known that the interaction mainly requires β-1,3-galactan chains, which are longer than five residues [21]. Thus, the purified material obtained after the extraction procedure using Yariv reagent, i.e. AGP, is mainly composed of glycan epitopes recognized by anti-AGP antibodies. Multiple ELISA tests performed allowed to get information about the variability of constantly occurring modifications of the carbohydrate moiety of AGPs which are correlated with the progress of the ripening process. From the data obtained, it can be assumed that the LM2 and LM14 epitopes are the main components of the sugar chains of AGPs present during the whole ripening program.

However, in the present paper, the use of immunocytochemical assays was intended to explain the results of the microbiological experiments. In previous studies, the high level of glycosylation of AGPs was considered to be the main determinant of AGP functions [4, 5]. Similarly, studies on the effect of AGPs from root caps on zoospore behavior and development in the pea-*Aphanomyces euteiches* pathosystem revealed the functional significance of the carbohydrate moiety of AGPs [36]. It is well-known that AGPs are involved in early root infection through selective induction of chemotaxis, zoospore encystment, and significant inhibition of cyst germination. The role of AGPs in the root-zoospore interaction was analyzed in comprehensive studies including biochemical assays, which confirmed quite substantial amounts of AGPs in the root border and border-like cells that are directly connected with key events in the establishment of infection. Also, by assaying chemotaxis, the authors proved that AGP fractions are effective in chemotactic response by attracting zoospores and may provide a chemical barrier to *A. euteiches* and immobilize the oomycete. These studies underlined the participation of AGPs in the inhibition of pathogen progression in the root-microbe interaction and highlighted the complexity of the molecular and cellular mechanism of interactions between plants and microbes [36]. Also in other studies, incubation with Yariv reagent caused a reduction of rhizobacterial colonization in the *Arabidopsis* root cap, indicating an essential role of AGPs in the cell protective mechanism [37]. The specific AGP glyco-structure was confirmed in our experiment, and we assumed that extensive glycosylation is crucial in the functional analysis of AGPs. Moreover, studies on AGPs isolated from fruit at different stages of the ripening process show the variability in the structure of their carbohydrate chains. The performed glycome profiling indicates that particular sugar chains of AGPs undergo modifications, with the predominance of degradation processes, which are most clearly noted in the last stages of the ripening process. Here, in both assays, i.e. the Antifungal (well-diffusion) Susceptibility Test (AFST) and the Agar Invasion Test (AIT), no impact of AGPs on *Penicillium* growth was noted. No effect was also observed in fractions containing a considerable quantity of purified AGPs. We may assume that even an increase in AG type II in AGPs extracted from the Mature Red fruit has no effect on the growth of the fungus. Unfortunately, AGP from fruit from every single stage of the ripening process had no effect on the *Penicillium* growth. Similarly, the purification and separation of the three main AGP fractions were aimed at verifying whether individual epitopes forming the sugar chain may have different properties with respect to fungal activity. However, in this case, similarly, none of the three fractions has no positive results in microbiological assays. All this information allows the formulation of a general conclusion: the cell response to fungal infection depends on the interaction in the entire plant cell. The action of a single component does not elicit any influence ex situ*.*

However, it has already been found that AGPs act as attractants for symbiotic species, promoting the development of infection structures and triggering the induction of alterations *in planta* [37]. Also, AGP degradation products, such as glycopeptides or oligosaccharides, are modulators of the plant immune system by preventing infections as damage-associated molecular patterns (DAMPs), which induce systemic resistance [38, 39]. Carbohydrate components (AG type II) released from AGPs by hydrolytic enzymes in infected tissue play a role in monitoring the stability of the cell wall by activation of response to damage [40]. Along the same lines, an increase in the abundance of AGPs in response to *Fusarium oxysporum* [41] and *Colletotrichum gloeosporioides* attack [42] was considered as a response to the maintenance of cell integrity and reinforcement of the cell wall [18]. The present study excludes AGP-pathogen interactions outside the cell in in vitro experimental conditions. On the other hand, the extensive accumulation of AGPs in infected tissue [19] as a result of a complex mechanism occurring in the cell wall emphasizes the importance of interactions between the components of the extracellular matrix. Hence, the absence of an AGP inhibitory effect on the fungus growth does not rule out their contribution to plant defense by restraining pathogens.

## Conclusion and perspective of the research

**Fig. 8:**
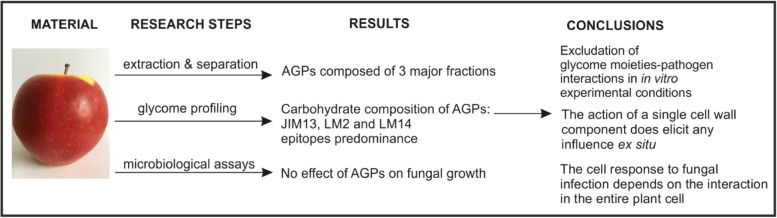
Summary of current work. Extraction of AGPs, glycome profiling, and microbiological assays allow concluding that carbohydrate chains of AGPs have no impact on fungal growth in in vitro conditions

Based on our results summarized in Fig. 8 and referring to recent reports about interactions of AGPs with other cell wall components, studies on mutants with a disorder of AGP synthesis should be undertaken in the future. Research on mutant backgrounds will allow investigation of whether the structurally altered AGPs have the same effect in situ on the progress of fungal infection. Concurrently, it clearly indicates the necessity to undertake further research aimed at examining the structural dependencies occurring in the plant cell wall. In addition, the study is required to determine the effect of the AGP molecular disorder on interlinking with other constituents of the extracellular matrix and thus its impact on the functional role in pathogen interaction.

## Supplementary Information

**Additional file 1.** RAW data of SDS-PAGE and Western blot analysis of fraction 1 (F1), fraction 2 (F2), and fraction 3 (F3). AGPs were detected by JIM13, JIM15, LM2 and LM14 antibodies.

**Additional file 2.** ELISA test of AGPs.

## Declarations

### Availability of data and material

The datasets analysed during the current study are available from the corresponding author on reasonable request.
