# Supplementary figures and images for: Working towards arabinogalactan proteins (AGPs) from fruit: carbohydrate composition and impact on fungal growth
Source: BMC Plant Biol. 2022 Dec 20;22:600. doi: 10.1186/s12870-022-04009-6 (PMC9764746; doi:10.1186/s12870-022-04009-6)

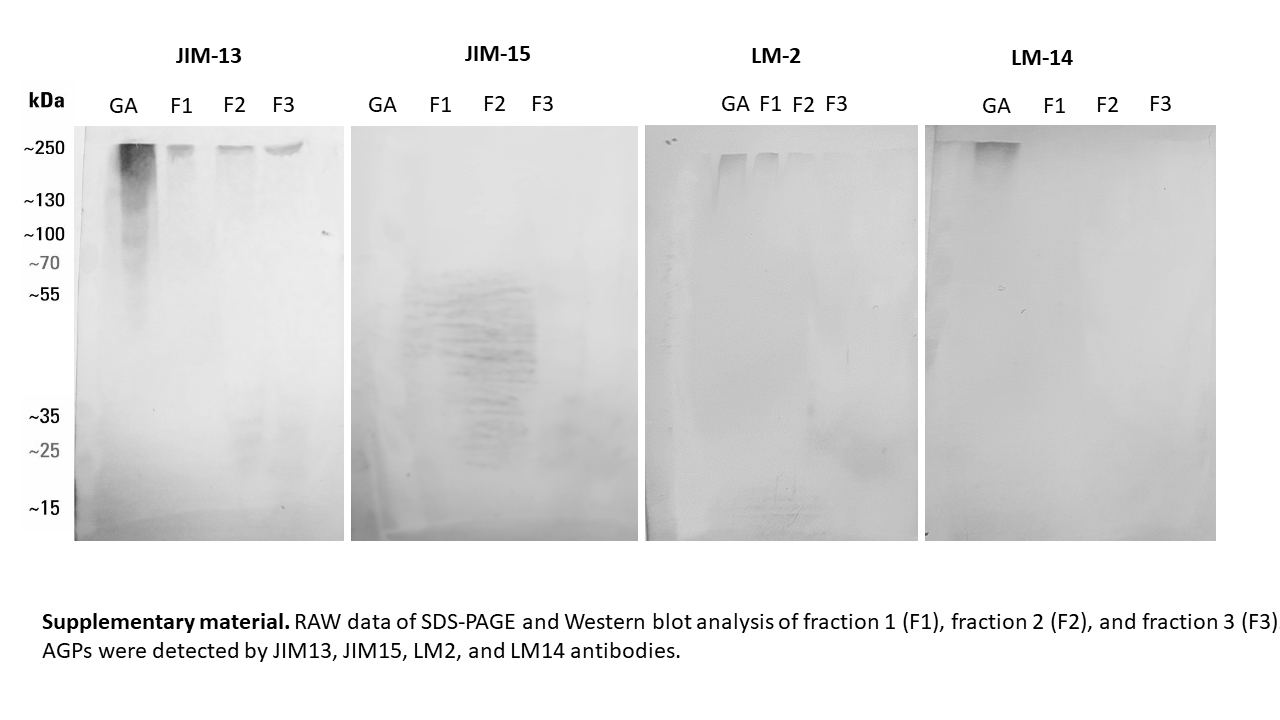

Supplement: Supplementary file 1 — Additional file 1. RAW data of SDS-PAGE and Western blot analysis of fraction 1 (F1), fraction 2 (F2), and fraction 3 (F3). AGPs were detected by JIM13, JIM15, LM2 and LM14 antibodies. [file 12870_2022_4009_MOESM1_ESM.tif]
