# Supplementary material for: Working towards arabinogalactan proteins (AGPs) from fruit: carbohydrate composition and impact on fungal growth
Source: BMC Plant Biol. 2022 Dec 20;22:600. doi: 10.1186/s12870-022-04009-6 (PMC9764746; doi:10.1186/s12870-022-04009-6)
Supplement: Supplementary file 2 — Additional file 2. ELISA test of AGPs. [file 12870_2022_4009_MOESM2_ESM.pdf]

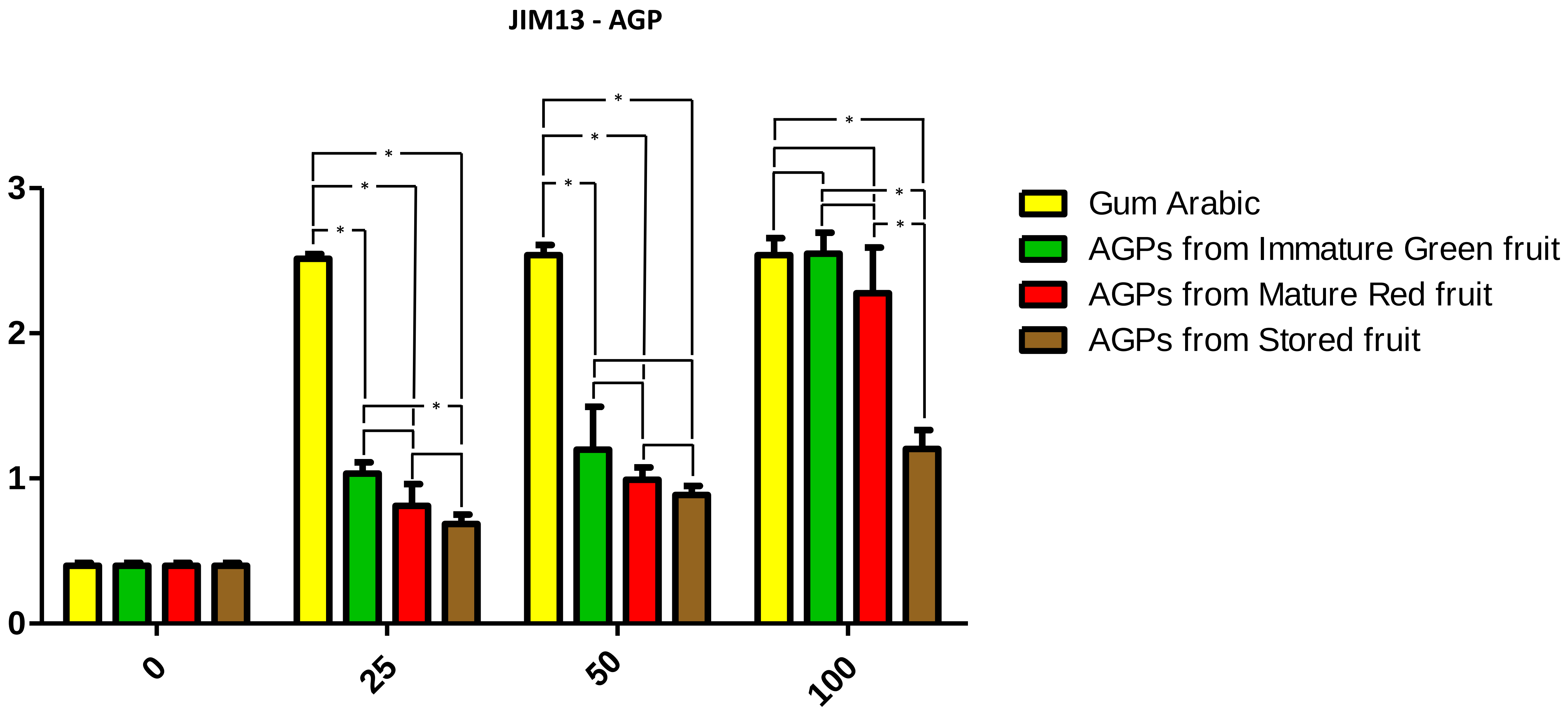

Supplementary material. ELISA test of AGPs from Immature Green fruit, AGPs from Mature Red fruit and AGPs from Stored fruit. AGPs were detected by JIM13 antibody. Gum Arabic was used as a positive control. For the analysis of variance (one-way ANOVA) followed by post hoc Tukey's honestly significant difference (HSD) test was used.

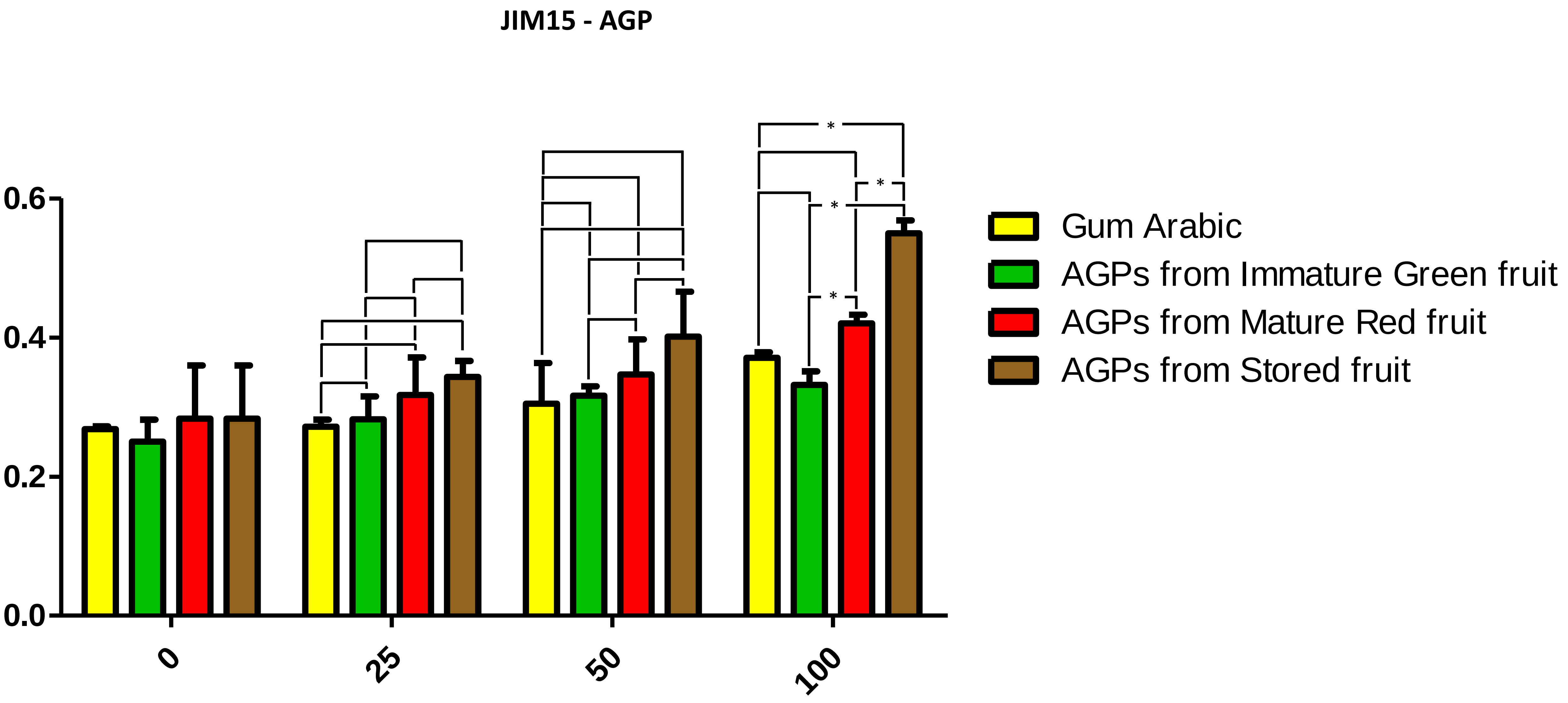

Supplementary material. ELISA test of AGPs from Immature Green fruit, AGPs from Mature Red fruit and AGPs from Stored fruit. AGPs were detected by JIM15 antibody. Gum Arabic was used as a positive control. For the analysis of variance (one-way ANOVA) followed by post hoc Tukey's honestly significant difference (HSD) test was used.

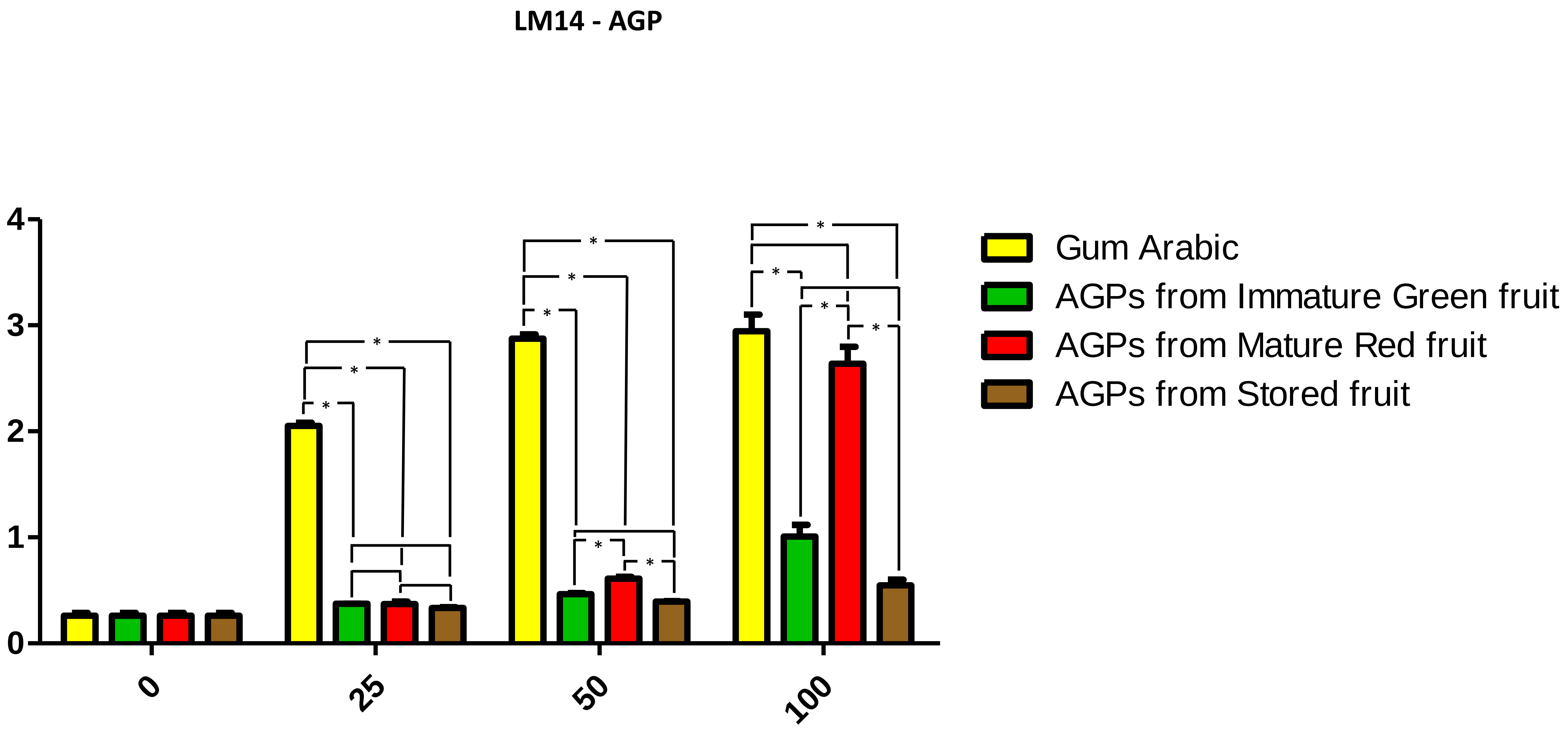

Supplementary material. ELISA test of AGPs from Immature Green fruit, AGPs from Mature Red fruit and AGPs from Stored fruit. AGPs were detected by LM14 antibody. Gum Arabic was used as a positive control. For the analysis of variance (one-way ANOVA) followed by post hoc Tukey's honestly significant difference (HSD) test was used.

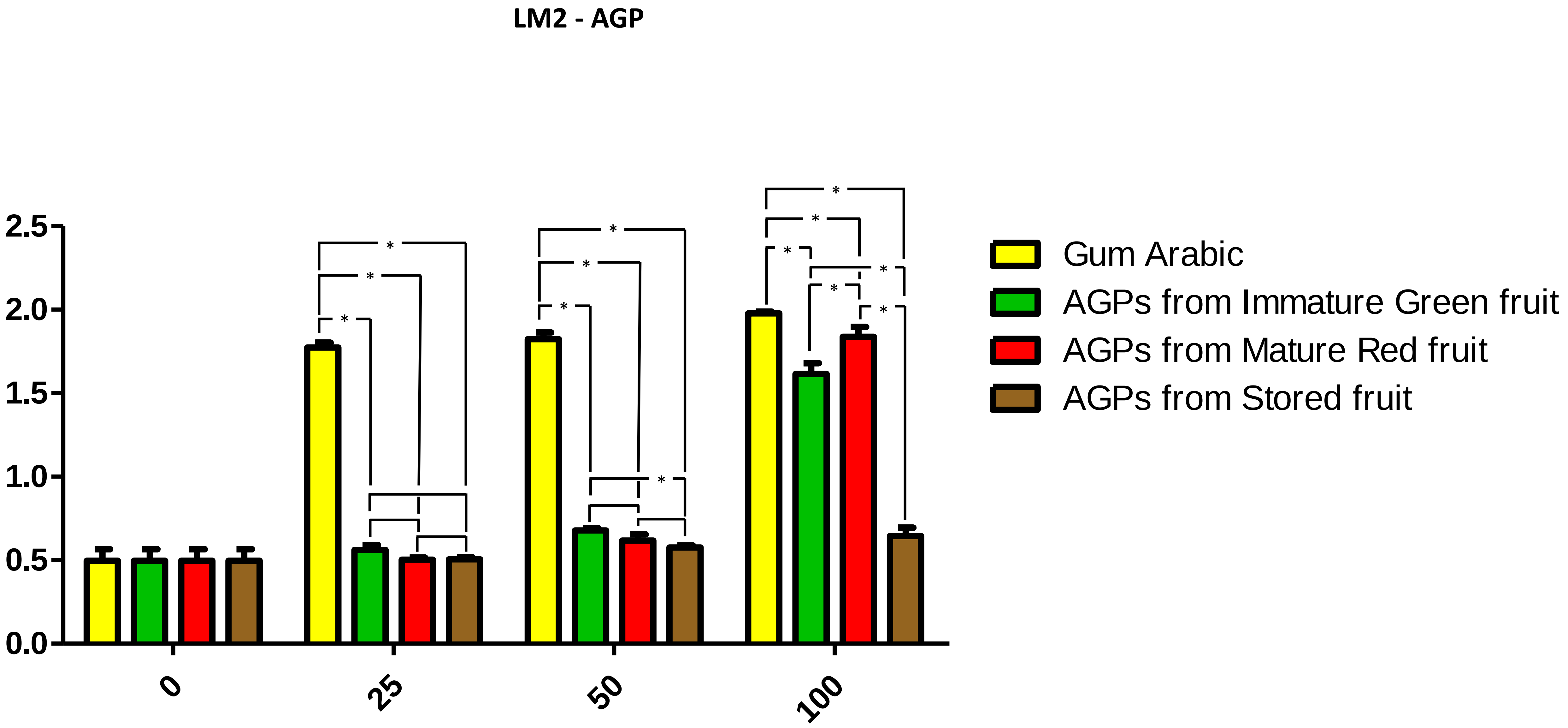

Supplementary material. ELISA test of AGPs from Immature Green fruit, AGPs from Mature Red fruit and AGPs from Stored fruit. AGPs were detected by LM2 antibody. Gum Arabic was used as a positive control. For the analysis of variance (one-way ANOVA) followed by post hoc Tukey's honestly significant difference (HSD) test was used.

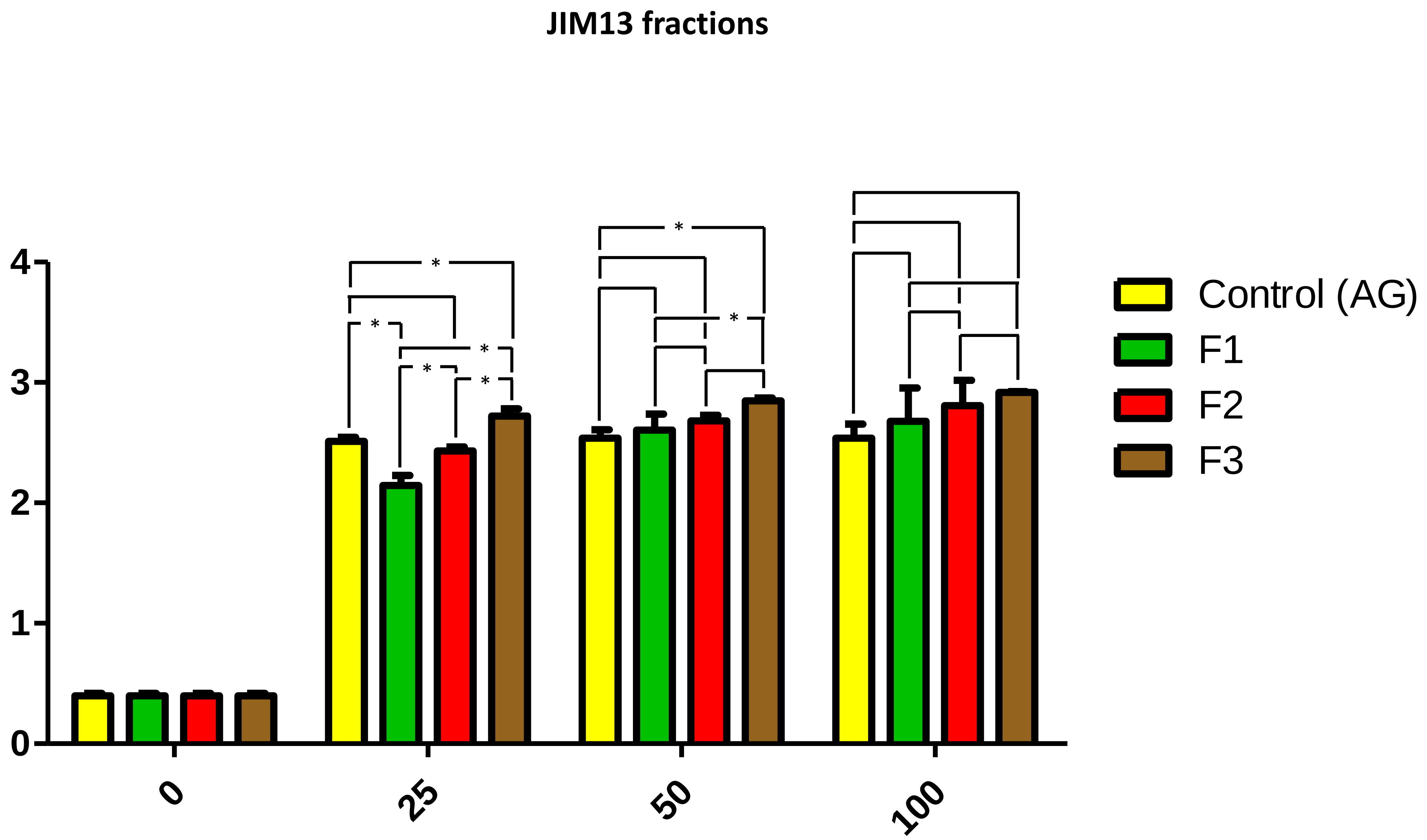

Supplementary material. ELISA test of fraction 1 (F1), fraction 2 (F2), and fraction 3 (F3). AGPs were detected by JIM13 antibody. Gum Arabic was used as a positive control. For the analysis of variance (one-way ANOVA) followed by post hoc Tukey's honestly significant difference (HSD) test was used.

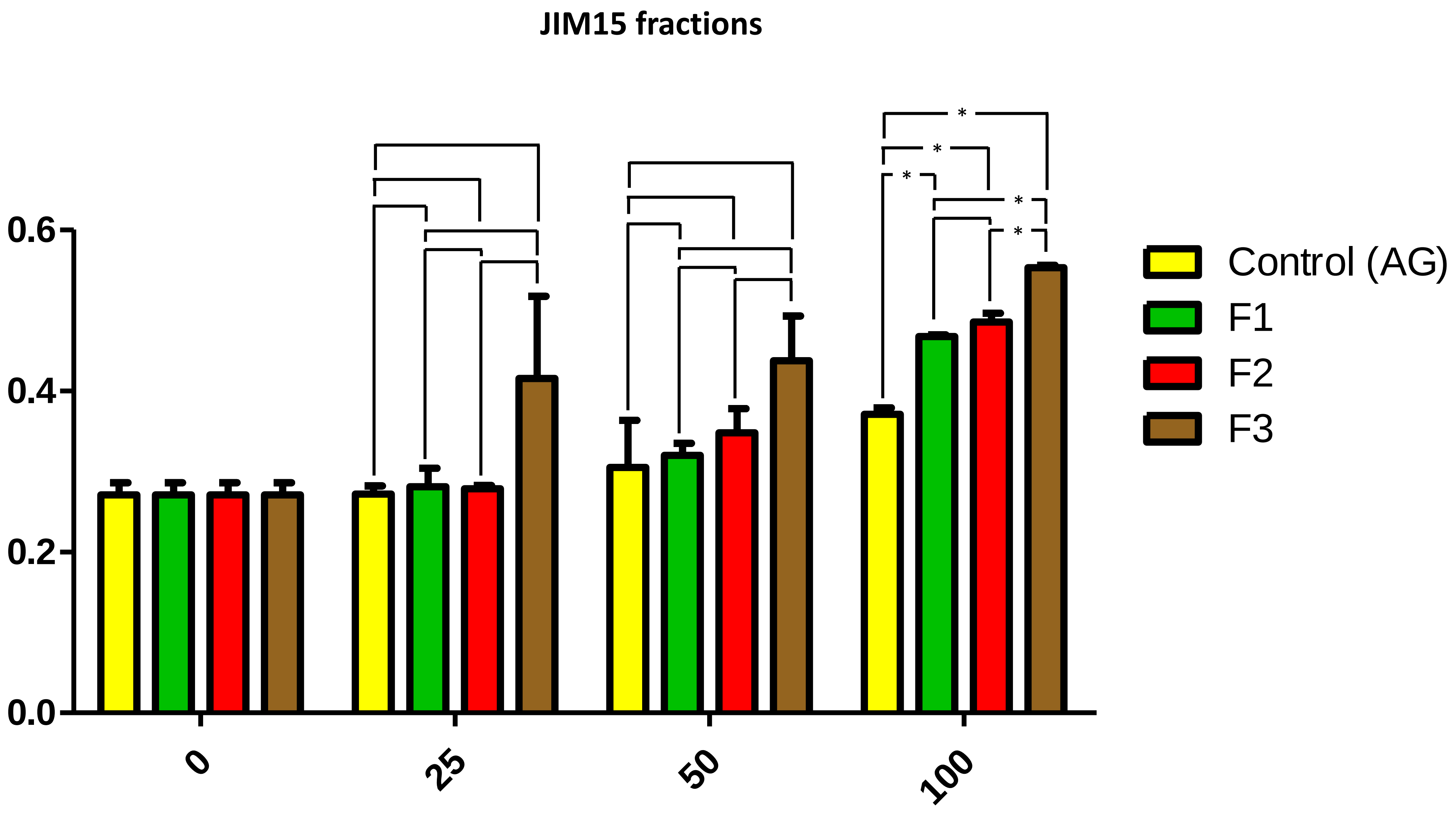

Supplementary material. ELISA test of fraction 1 (F1), fraction 2 (F2), and fraction 3 (F3). AGPs were detected by JIM15 antibody. Gum Arabic was used as a positive control. For the analysis of variance (one-way ANOVA) followed by post hoc Tukey's honestly significant difference (HSD) test was used.

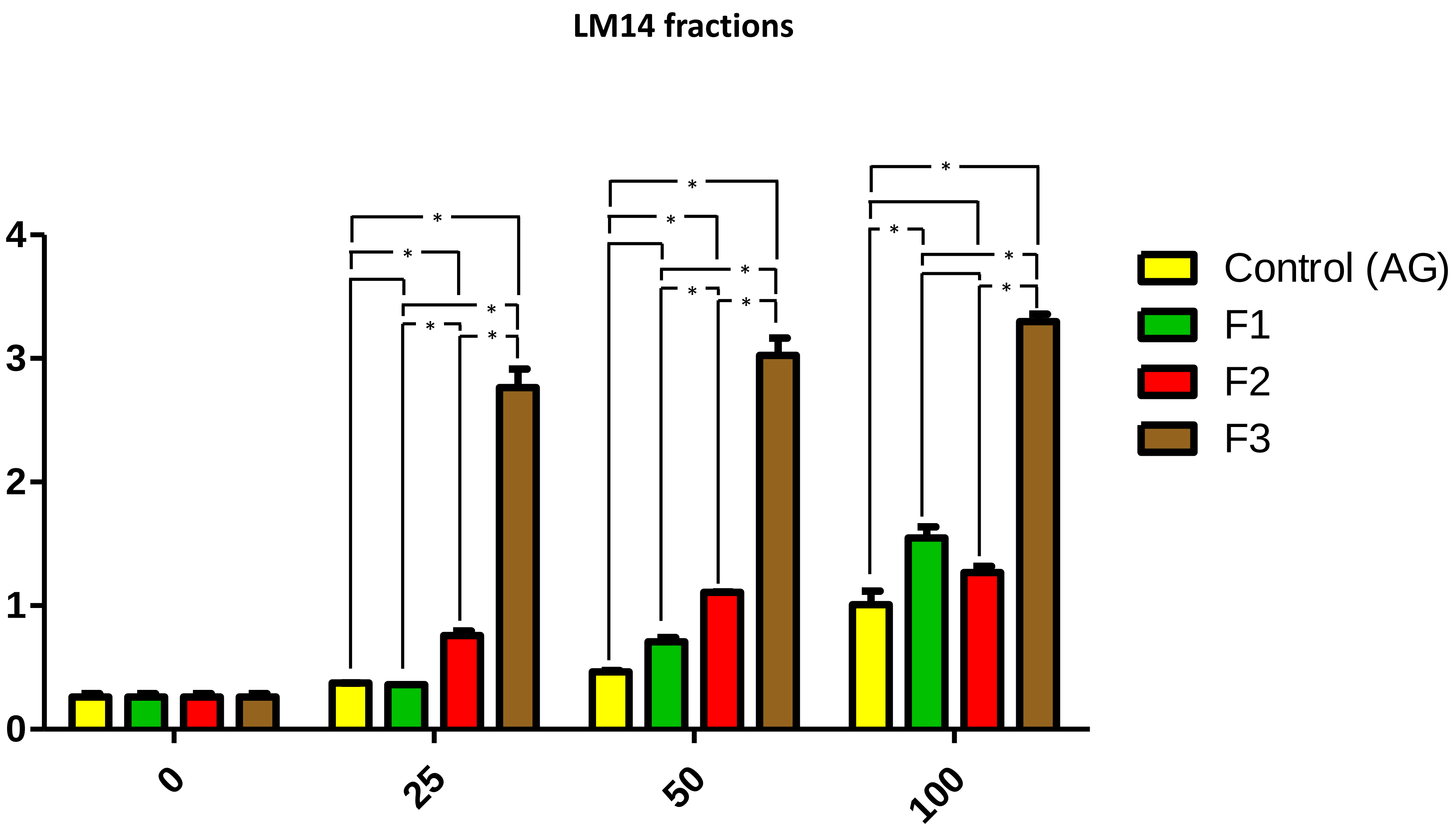

Supplementary material. ELISA test of fraction 1 (F1), fraction 2 (F2), and fraction 3 (F3). AGPs were detected by LM14 antibody. Gum Arabic was used as a positive control. For the analysis of variance (one-way ANOVA) followed by post hoc Tukey's honestly significant difference (HSD) test was used.

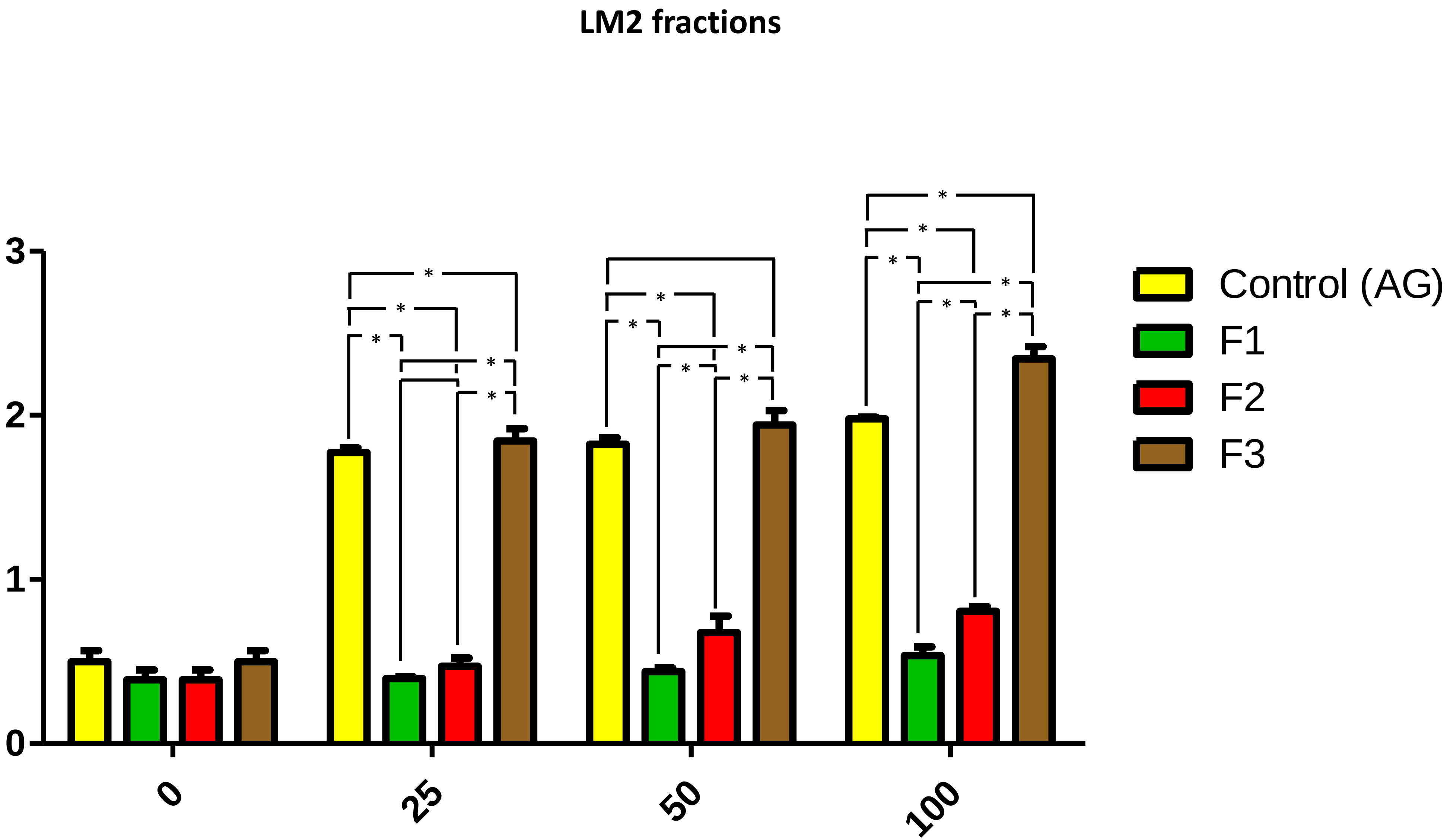

Supplementary material. ELISA test of fraction 1 (F1), fraction 2 (F2), and fraction 3 (F3). AGPs were detected by LM2 antibody. Gum Arabic was used as a positive control. For the analysis of variance (one-way ANOVA) followed by post hoc Tukey's honestly significant difference (HSD) test was used.
